# Impact of the frequency of online verifications on the patient set-up accuracy and set-up margins

**DOI:** 10.1186/1748-717X-6-101

**Published:** 2011-08-24

**Authors:** Volker Rudat, Mohamed Hammoud, Yogin Pillay, Abdul Aziz Alaradi, Adel Mohamed, Saleh Altuwaijri

**Affiliations:** 1 Department of Radiation Oncology, Saad Specialist Hospital, P.O. Box 30353, Al Khobar 31952, Saudi Arabia; 2 SAAD Research & Development Center, Saad Specialist Hospital, P.O. Box 30353, Al Khobar 31952, Saudi Arabia

## Abstract

**Purpose:**

The purpose of the study was to evaluate the patient set-up error of different anatomical sites, to estimate the effect of different frequencies of online verifications on the patient set-up accuracy, and to calculate margins to accommodate for the patient set-up error (ICRU set-up margin, SM).

**Methods and materials:**

Alignment data of 148 patients treated with inversed planned intensity modulated radiotherapy (IMRT) or three-dimensional conformal radiotherapy (3D-CRT) of the head and neck (n = 31), chest (n = 72), abdomen (n = 15), and pelvis (n = 30) were evaluated. The patient set-up accuracy was assessed using orthogonal megavoltage electronic portal images of 2328 fractions of 173 planning target volumes (PTV). In 25 patients, two PTVs were analyzed where the PTVs were located in different anatomical sites and treated in two different radiotherapy courses. The patient set-up error and the corresponding SM were retrospectively determined assuming no online verification, online verification once a week and online verification every other day.

**Results:**

The SM could be effectively reduced with increasing frequency of online verifications. However, a significant frequency of relevant set-up errors remained even after online verification every other day. For example, residual set-up errors larger than 5 mm were observed on average in 18% to 27% of all fractions of patients treated in the chest, abdomen and pelvis, and in 10% of fractions of patients treated in the head and neck after online verification every other day.

**Conclusion:**

In patients where high set-up accuracy is desired, daily online verification is highly recommended.

## Introduction

Linear accelerators capable of image-guided radiotherapy (IGRT) have become available in a large number of institutions. With the new on-board imaging technologies, patient positioning verification has become more accurate [1,2]. IGRT also offers the opportunity of frequent online treatment verification in the clinical routine, which may lead to modifications of verification protocols popular in the pre-IGRT era.

The frequency of online verifications should generally be as low as necessary to achieve the desired patient positioning accuracy in order to save machine-time and imaging dose to the patient. At the same time, the safety margin to accommodate for the patient positioning error should be as small as possible in order to reduce the dose to normal tissue.

The International Commission on Radiation Units and Measurements (ICRU) has defined two margins to compensate for geometric variation and uncertainties that may impede the exact delivery of a treatment plan: The Internal margin (IM) and set-up margin (SM). The IM accounts for expected organ motion and deformation, and the SM for patient set-up errors due to variations in the daily positioning of the patient on the treatment couch. Mechanical uncertainties of the equipment (*e.g*., sagging of the couch), dosimetric uncertainties, transfer set-up errors from CT-Simulator to the treatment unit, and human related errors also contribute to the SM. The planning target volume (PTV) encompasses the clinical target volume (CTV), the IM, and SM.

In this study we measured the set-up error of patients treated in the head and neck region, chest, abdomen, and pelvis by using electronic portal imaging. In addition, the effect of different frequencies of online verification (no online verification, online verification once a week, online verification every other day) on the patient set-up error was evaluated, and for each scenario the corresponding SM calculated.

The data should help the physician to choose the most clinically appropriate frequency of online verification for the individual patient by balancing the "cost" of online verification (machine-time and imaging dose to the patient) with the risk of radiation toxicity related to the size of the PTV.

## Methods and materials

One hundred and forty-eight patients treated with inversed planned intensity modulated radiotherapy (IMRT) or three-dimensional conformal radiotherapy (3D-CRT) of the head and neck (n = 31), chest (n = 72), abdomen (n = 15), and pelvis (n = 30) were evaluated. Patients treated in a belly board were excluded from the analysis because the set-up error in prone position has been shown to be significantly larger compared to supine position [3,4]. The patient set-up error was assessed using orthogonal electronic portal images of 2328 fractions of 173 planning target volumes (PTV). In 25 patients, two PTVs were analyzed where the PTVs were located in different anatomical sites and treated in two different radiotherapy courses. Electronic portal images were taken daily of all patients where a high dose was prescribed and organs at risk were located in close proximity to the PTV (all patients treated with IMRT and selected patients treated with 3D-CRT; n = 60 [35%]). For all other patients, electronic portal images were taken on days 1-3, then every other day (n = 113 [65%]). The average number of fractions with electronic portal images per PTV was 13, and the range was 3 to 43.

### Patient immobilization and treatment planning

All patients treated in the head and neck region were immobilized using a thermoplastic mask in a carbon frame, and a kneefix. Patients treated in the chest were immobilized using a Silverman headrest, wing board or C-Qual breastboard, and a kneefix. Patients treated in the abdomen or pelvis were immobilized using a Silverman headrest, kneefix, and feetfix. The CT-Simulator, the PET-CT, and the linear accelerators were equipped with identical models of a carbon index tables, and positioning devices (CIVCO, Iowa, U.S.A.). The CT-simulator and the PET-CT were equipped with red lasers, the linear accelerators with green lasers.

CT-Simulation was performed using a CT Simulator (Somatom Sensation Open, Siemens Medical, Germany) or PET-CT (Biograph 64, Siemens Medical, Germany). The slice thickness was 3 mm or 5 mm. The CT scanning reference point and target volumes (PTV and organs at risk) were defined using specific software (Coherence Therapist and Coherence Oncologist, Siemens Medical, Germany). The IMRT and 3D-CRT plans were generated using the treatment planning system XIO 4.4 (CMS, Inc. of St. Louis, Mo, USA). Linear accelerators (Oncor Avant Garde, Siemens Medical, Germany) with dual photon energy of 6 MV and 15 MV, multileaf collimator (80 leaves, after upgrade 160 leaves), and EPID (Optivue, Siemen Medical, Germany) were used for the treatment.

### Online treatment verification

Orthogonal megavoltage electronic portal images were generated prior to treatment. Processing and analysis software was used to significantly improve the image quality of the megavoltage electronic portal images [1]. Representative bony landmarks as recommended by the report "On target: ensuring geometric accuracy in radiotherapy" by The Royal College of Radiologists [5] and in addition the trachea in chest patients [6] were marked using electronic drawing tools and compared with corresponding digitally reconstructed radiographs generated by the treatment planning system. Images were zoomed and electronically superposed. The portal imaging software calculated the deviation of the corresponding isocenters. Online correction was done by automatic adjustment of the treatment table in three dimensions prior to treatment. Repeated portal images were taken after table correction for the first 20 patients. Thereafter, this practice was discontinued because the automatic table correction showed to be consistently precise.

### Statistical Analysis

Individual and population based patient positioning accuracy parameters were calculated according to the report "On target: ensuring geometric accuracy in radiotherapy" by The Royal College of Radiologists [5]. Accordingly, the individual mean set-up error M_individual _was defined as the mean set-up error for an individual patient. The overall population mean set-up error M_pop _was defined as the overall mean for the analyzed patient group. The population systematic error Σ_set-up _was defined as the standard deviation of the individual mean set-up error about the overall mean M_pop_. The individual random (daily) set-up error σ_individual _was defined as the standard deviation of the set-up error around the corresponding mean individual value M_individual_. The population random error σ_set-up _was defined as the mean of all individual random errors σ_individual_.

The patient set-up accuracy parameters for each direction (anteroposterior, lateral and superoinferior) were calculated for patients treated in the head and neck region, chest, abdomen, and pelvis separately. A multivariate analysis of variance (ANOVA) and the Bonferroni test for post-hoc comparison were performed to test for statistically significant differences of the systematic and random set-up error of patients treated in the different anatomical regions. For the ANOVA, M_individual _and σ_individual _were used as dependent variables, the anatomical region (head and neck, chest, abdomen, and pelvis), and the direction (anteroposterior, lateral, and superoinferior) as categorical factors.

In order to estimate the patient set-up accuracy without online verification, online verification once per week, and online verification every other day, the patient set-up parameters were retrospectively calculated assuming a patient set-up error of 0 mm in all directions after online correction. Due to possible hardware and software related inaccuracies, the true set-up error after online correction will be more than 0 mm. However, phantom measurements assessing the precision of laser alignments in our department showed that all deviations of the reference point at the linear accelerator compared to the CT simulation reference point were below 1 mm (data not shown).

Treatment margins were calculated using the van Herk formula [7]. Accordingly, the margin required to ensure 95% minimum dose to the PTV for 90% of the patients was given by:

(1) $$M_{ptv}=2.50\Sigma +1.64\sigma -1.64\sigma_{P}$$

where Σ is the square-root of the quadratic sum of the standard deviations of all contributing systematic errors, σ the square-root of the quadratic sum of the standard deviations of all contributing random errors, and *σ_P _*the standard deviation describing the width of the penumbra. In our analysis *Σ_set-up _*was used as contributing systematic error, and *σ_set - up _*and *σ_P _*as contributing random errors$\left(\sigma =\sqrt[{2}]{\sigma_{set-up}^{2}+\sigma_{P}^{2}}\right)$. The organ motion, transfer and delineation errors were not considered in the calculation of the treatment margins because the focus of this study was the patient positioning set-up error. The representative standard deviation of the penumbra width *σ_P _*of our linear accelerators was 4.2 mm.

Residual set-up errors were calculated as percentage of the total number of measurements above the specified cut-off. For the calculation of the residual error the three-dimensional vector of the set-up error was used.

## Results

The population based patient set-up parameters without online verification, online verification once a week, and online verification every other day are listed in table 1. The data show an effective improvement of both the systematic and the random error with increasing frequency of online verifications. The systematic error tended to be smaller than the random error in all scenarios and improved from no online verification to online verification every other day relatively more than the random error (on average by a factor of 2.1 versus 1.4).

**Table 1:** Patient set-up error (mm) for each scenario in three dimensions

		Direction								
		
		AP			Lateral			SI		
		
Anatomical region	FOV	M	Σ	σ	M	Σ	σ	M	Σ	σ
Head and Neck	0%	0.3	0.9	1.6	-0.3	1.3	1.6	0.6	1.5	2.2
Chest	0%	0.7	2.4	2.7	-0.3	2.2	2.7	0.5	1.7	2.4
Abdomen	0%	0.6	3.0	3.3	-0.9	2.4	3.0	-0.8	3.6	3.1
Pelvis	0%	0.9	2.3	3.2	-0.3	1.8	2.7	1.0	3.2	2.5
										
Head and Neck	20%	0.2	0.8	1.4	-0.3	1.1	1.4	0.4	1.1	1.9
Chest	20%	0.5	1.7	2.2	-0.3	1.7	2.4	0.3	1.4	2.1
Abdomen	20%	0.6	2.3	3.1	-0.5	1.6	2.7	-0.4	2.6	3.0
Pelvis	20%	0.6	1.7	2.9	-0.4	1.5	2.4	0.9	2.6	2.3
										
Head and Neck	50%	0.1	0.5	1.1	-0.1	0.6	1.2	0.2	0.6	1.3
Chest	50%	0.3	0.9	1.6	-0.2	1.1	2.0	0.2	0.8	1.6
Abdomen	50%	0.4	1.5	2.5	-0.2	1.1	2.5	-0.4	1.7	2.8
Pelvis	50%	0.5	1.3	2.4	-0.2	1.1	1.9	0.6	1.4	2.0

*Abbreviations: *M = Overall population mean set-up error; Σ = Population systematic error; σ = Population random error; AP = anteroposterior; SI = superoinferior; FOV = Frequency of online verifications; 0% = No online verification; 20% = Online verification once a week; 50% = Online verification every other day.

An ANOVA with the Bonferroni test for post-hoc comparison of the patient set-up parameters without online verification showed a significantly smaller patient set-up random error for patients treated in the head and neck compared to the patients treated in the chest, abdomen, or pelvis (p < 0.01). This result was most probably due to the more effective patient positioning immobilization by mask fixation. No significant different patient set-up random error was found between the patients treated in the chest, abdomen, or pelvis in the three directions: anteroposterior, lateral, and superoinferior. A small but significant difference of the patient mean set-up error was found in the lateral direction (-0.50 mm) compared to the anteroposterior (0.58 mm) or superoinferior (0.39 mm) direction (p < 0.01).

**Figure 1:**
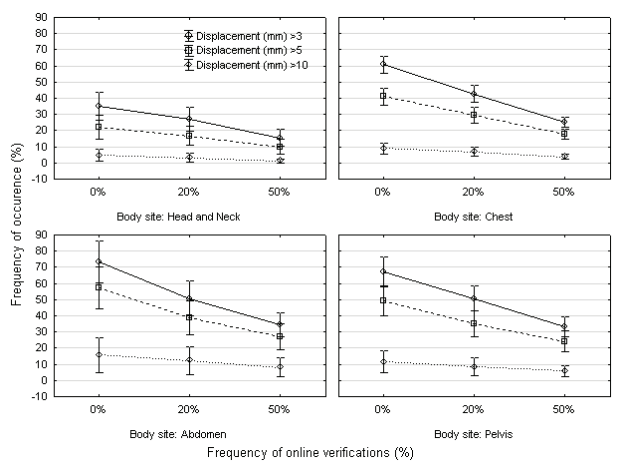
**Frequency of set-up errors larger than threshold (three-dimensional vector) for all scenarios and all fractions**. The frequency of online verifications is plotted on the horizontal axis (0%, no online verification; 20%, online verification once a week; 50%, online verification every other day), and the percentage of fractions with set-up errors larger than 3 mm, 5 mm or 10 mm on the vertical axis.

**Figure 2:**
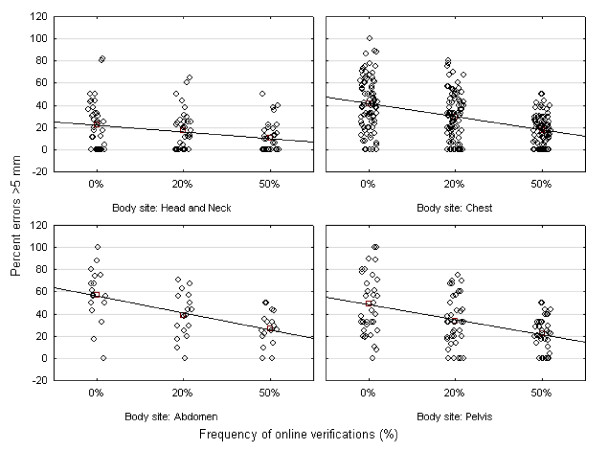
**Interindividual variability of frequencies of set-up errors larger than 5 mm (three-dimensional vector)**. The frequency of online verifications is plotted on the horizontal axis (0%, no online verification; 20%, online verification once a week; 50%, online verification every other day), and the percentage of fractions with set-up errors larger 5 mm on the vertical axis.

Figure 1 shows the frequency of set-up errors larger than 3 mm, 5 mm, and 10 mm of patients treated in the head and neck, chest, abdomen, and pelvis. A considerable frequency of relevant residual set-up errors even after online verification every other day was demonstrated. The marked interindividual variability of the frequency of residual errors larger than 5 mm is demonstrated in Figure 2.

The mean time for online verification (acquisition of orthogonal portal images and set-up correction before treatment if necessary) was 3.6 minutes per fraction (standard deviation 0.5 minutes), and on average 4 monitor units per fraction were applied for the portal imaging.

Table 2 shows that the SM calculated using the van Herk formula [7] decreased with increasing frequency of online verification.

**Table 2:** Set-up margins (mm) for each scenario using the van Herk formula [3]

		Safety Margin*		
		
Anatomical region	FOV	AP	Lateral	SI
Head and Neck	0%	3	4	5
Chest	0%	7	7	5
Abdomen	0%	9	8	11
Pelvis	0%	8	6	9
				
Head and Neck	20%	2	3	3
Chest	20%	5	5	4
Abdomen	20%	7	5	8
Pelvis	20%	6	5	7
				
Head and Neck	50%	1	2	2
Chest	50%	3	3	2
Abdomen	50%	5	4	6
Pelvis	50%	4	3	4

Abbreviations: * = 95% of the dose for 90% of the patients; other abbreviations as in Table 1.

## Discussion

The purpose of this study was to evaluate the patient set-up error of different anatomical sites, to estimate the effect of different frequencies of online verifications on the patient set-up accuracy, and to calculate the corresponding SM.

Our data show that the patient set-up error improved effectively with increasing frequency of online verification, but that a considerable frequency of relevant set-up errors remained even after online verification every other day. For example, residual set-up errors larger than 5 mm were observed on average in 18% to 27% of all fractions of patients treated in the chest, abdomen and pelvis, and in 10% of fractions of patients treated in the head and neck after online verification every other day. The higher set-up accuracy of the head and neck region was most probably due to the more effective immobilization using the mask fixation. We conclude that less than daily online verification may lead to suboptimal results in patients where high set-up accuracy is desired. Another observation supporting this conclusion is the marked interindividual variability of the patient set-up accuracy. This may result in a treatment with clinically unacceptable high frequency of set-up errors larger than 5 mm in individual patients if population based safety margins are used and online verification is done less than daily. For example, the patient with the worst patient positioning accuracy in our study had a frequency of displacements larger than 5 mm in 50% of all fractions after online verification every other day.

The frequency of set-up errors above a certain level that can be tolerated is a clinical decision involving factors associated with prognosis, risk of failure, and toxicity. The calculation of the safety margin based on the patient set-up accuracy after different frequencies of online verifications would enable the radiation oncologist to select the most appropriate approach in terms of size of the PTV versus cost associated with imaging in terms of in-room time and imaging dose to the patient. In our institution we decided to perform daily online verifications in all patients treated with IMRT and in patients treated with 3D-CRT where a high dose is prescribed and critical organs at risk are located in close proximity to the PTV. Patients, for example, where the prescribed dose does not exceed the tolerance dose of relevant organs at risk may be treated with a lower frequency of online verifications and with a correspondingly larger PTV. The "cost" of in-room time observed in our study of 3.6 minutes (standard deviation 0.5 minutes) per patient and fraction was considered acceptable. Furthermore, imaging dose to the patient is minimal if portal imaging is used compared to cone-beam computed tomography (CBCT), and lower if kilovoltage X-rays are used compared to megavoltage X-rays [8].

We analyzed the patient set-up accuracy using the concept of systematic and random errors. The systematic component of any errors can be defined as a deviation that occurs in the same direction and is of a similar magnitude for each fraction throughout the treatment course ("treatment preparation errors"), and the random component as a deviation that can vary in direction and magnitude for each delivered treatment fraction ("treatment execution errors"). The differentiation between systematic and random errors is not only important to identify sources of errors, it is also important for the derivation of appropriate safety margins. Typically the key contributor to the margin is the combined systematic error. Using the van Herk formula [7] with the assumption to cover the PTV with ≥95% of the prescribed dose in 90% of the patients, the SM of the different anatomical sites and directions could be reduced from 3-11 mm without online verification to 1-6 mm after online verification every other day. It should be noted that these safety margins have to be considered as minimum margins because the delineation error, transfer error, and organ motion were not considered in our analysis. In addition, possible rotational errors and changes of the shape of the tumor during radiotherapy are ignored by the van Herk model [7].

The systematic and random patient set-up errors observed in our study are well in line with corresponding published reports [3,9-33]. The impact of daily online verification on the PTV has been extensively studied in patients treated with definitive radiotherapy for prostate cancer. For this tumor entity, the target positioning accuracy is of paramount importance because of the high dose prescribed, the close proximity of the organs at risk: bladder and rectum to the prostate, and the use of the highly conformal treatment technique IMRT. Image-guided radiotherapy (IGRT) using fiducial prostate markers, in-room CT, or cone-beam CT were used to control for the prostate motion. All reports showed that daily online verification permits the use of narrower CTV-PTV margins without compromising coverage of the target [23,25-32]. Kupelian et al. retrospectively compared different image-guided strategies in the alignment of prostate cancer patients using fiducial prostate gold markers. The authors showed that the systematic error was effectively reduced with imaging, but that the magnitude of random errors remained unaffected at the treatment sessions not associated with image guidance. In line with our results, a significant frequency of relevant residual errors was found even after online verification every other day, and the authors suggested that daily localizations should be performed in the set-up of prostate cancer patients during a course of external beam radiotherapy [27].

The focus of our analysis was the patient set-up accuracy. Geometric uncertainties due to organ motion were not analyzed in this study. Therefore an IM has to be added to the SM proposed in our study to define the PTV [7,34].

However, the ultimate goal would be to achieve the planned dose distribution. The most precise approach to accomplish this goal would be the use of daily kilovoltage CT-based online verification with excellent soft-tissue image quality, delineation of all relevant structures, and online recalculation of plan parameters if necessary [2]. Technologies are currently under development that will allow this approach in a time and workflow feasible for clinical routine application.

## Conclusions

The ICRU set-up margin (SM) could be reduced with increasing frequency of online verification but a considerable frequency of relevant set-up errors remain even after online verification every other day. For example, residual set-up errors larger than 5 mm were observed on average in 18% to 27% of all fractions of patients treated in the chest, abdomen, and pelvis, and in 10% of fractions of patients treated in the head and neck after online verification every other day. We conclude that in patients where high set-up accuracy is desired, daily online verification is highly recommended.

## List of abbreviations

3D-CRT: Three-dimensional conformal radiotherapy; ANOVA: Multivariate analysis of variance; EPID: Electronic portal imaging device; IGRT: Image-guided radiotherapy; IM: ICRU internal margin; IMRT: Inversed planned intensity modulated radiotherapy; PTV: Planning target volume; SM: ICRU set-up margin

## Competing interests

The authors declare that they have no competing interests.

## Authors' contributions

MH, YP, AA, and AM participated in the study design, contributed to the data collection, and helped to draft the manuscript. SA participated in its design and coordination and helped to draft the manuscript. VR conceived of the study, participated in its design and coordination, participated in the treatment panning, performed the statistical analysis, and drafted the manuscript. All authors read and approved the final manuscript.
